# Mediastinal Gray Zone Lymphomas: Diagnostic Challenges, Clinicopathologic Overlap, and Emerging Management Strategies

**DOI:** 10.3390/hematolrep18010005

**Published:** 2025-12-31

**Authors:** Tugba Zorlu, Mert Seyhan, Nigar Abdullayeva, Turgay Ulas, Mehmet Sinan Dal

**Affiliations:** Department of Hematology & Apheresis Unit, Ankara Oncology Training and Research Hospital, University of Health Sciences, Ankara 06200, Türkiye; tbazrlu@gmail.com (T.Z.); drseyhanmert@gmail.com (M.S.); dr.sinandal@gmail.com (M.S.D.)

**Keywords:** brentuximab vedotin, PD-1 inhibitors, immunophenotype, molecular features, prognosis, treatment

## Abstract

**Background:** Mediastinal gray zone lymphoma (MGZL) is a rare B-cell lymphoma characterized by overlapping clinicopathologic and molecular features of primary mediastinal B-cell lymphoma (PMBL) and classical Hodgkin lymphoma (CHL). Under current WHO-HEMA5 and International Consensus Classification (ICC) frameworks, MGZL is restricted to EBV-negative lymphomas arising in the mediastinum. **Methods:** This review summarizes current evidence on epidemiology, clinical presentation, pathology, molecular characteristics, diagnostic challenges, and therapeutic approaches to MGZL, with data derived from retrospective series, limited prospective cohorts, and recent molecular studies. **Results:** MGZL predominantly affects young adults and commonly presents with bulky mediastinal disease. Diagnosis is challenging due to transitional morphology, pleomorphic Reed–Sternberg-like cells, and variable expression of B-cell and activation markers. Molecular studies demonstrate shared alterations with PMBL and CHL, including 9p24.1 (JAK2/PD-L1/PD-L2) gains, while additional reported features such as HOXA5 hypomethylation and MYC copy number gains support its biological distinctiveness, although evidence remains limited. Frontline treatment commonly involves intensive chemoimmunotherapy regimens such as DA-EPOCH-R; however, outcomes remain inferior to PMBL and CHL, with 5-year overall survival rates of approximately 40–60%. Relapsed or refractory disease frequently requires salvage chemotherapy and autologous stem cell transplantation. Immune-based therapies, including brentuximab vedotin and PD-1 inhibitors, have shown promising activity, particularly in combination. **Conclusions:** MGZL remains a diagnostically challenging and therapeutically complex lymphoma with inferior outcomes compared with related mediastinal lymphomas. Advances in molecular profiling and immunotherapy offer promising avenues toward more personalized treatment; however, prospective clinical trials and international collaboration are urgently needed to establish evidence-based management strategies for this rare entity.

## 1. Introduction

Mediastinal gray zone lymphoma (MGZL) is a distinct B-cell lymphoma of mediastinal origin characterized by overlapping clinicopathologic and immunophenotypic features of primary mediastinal B-cell lymphoma (PMBL) and classical Hodgkin lymphoma (CHL). According to the current WHO-HEMA5 and International Consensus Classification (ICC) frameworks, MGZL is restricted to EBV-negative lymphomas arising in the mediastinum, explicitly excluding extramediastinal and EBV-positive gray zone entities [1,2].

Since its recognition as a separate entity, MGZL has posed substantial diagnostic and therapeutic challenges owing to its rarity, morphologic and immunophenotypic heterogeneity, lack of standardized treatment algorithms, and its often-aggressive clinical behavior [1,2]. Earlier literature used the broader term gray zone lymphoma (GZL) to encompass biologically heterogeneous lymphomas spanning the spectrum between diffuse large B-cell lymphoma (DLBCL), particularly PMBL, and nodular sclerosis CHL [3]. However, many of these historical series included non-mediastinal and EBV-positive cases, complicating interpretation of diagnostic, molecular, and therapeutic data [4,5,6]. In this review, we therefore focus exclusively on strictly defined mediastinal, EBV-negative MGZL, summarizing current evidence on its epidemiology, clinical presentation, pathology, and treatment, while highlighting ongoing challenges and unmet clinical needs.

## 2. Historical Perspective and WHO Classification of MGZL

MGZL was initially recognized as a clinicopathologic entity in the late 1990s following reports of mediastinal lymphomas displaying intermediate features between DLBCL and CHL [7,8]. This concept led to the inclusion of a provisional category—“B-cell lymphoma, unclassifiable, with features intermediate between DLBCL and CHL”—in the 2008 WHO classification [2], which was retained in the 2017 revision. Subsequent molecular and clinicopathologic studies demonstrated that mediastinal cases are biologically distinct from extramediastinal gray zone lymphomas, with the latter being more closely related to DLBCL, not otherwise specified (NOS) [9,10,11]. These findings resulted in refinement of the entity in the WHO-HEMA5 (2022) and ICC (2022) classifications, which now restrict the diagnosis to mediastinal gray zone lymphoma, arising from thymic B cells and sharing features that are intermediate between PMBL and nodular sclerosis CHL [1,2]. EBV-positive lymphomas and composite or sequential CHL/PMBL presentations are explicitly excluded.

## 3. Clinical Presentation

MGZL predominantly affects young adults, with a median age in the mid-30s, and shows a marked male predominance, accounting for approximately two-thirds of cases [4,12]. The most common presentation is a bulky mediastinal mass, observed in nearly half of patients, often associated with symptoms related to local compression, including superior vena cava syndrome, dyspnea, or cough. At initial diagnosis, disease is frequently confined to the mediastinum, and stage IV disease is uncommon, reported in approximately 10–15% of cases [12].

Disease progression may involve locoregional extension to adjacent thoracic structures or, less commonly, secondary involvement of extranodal sites such as the liver, spleen, or bone marrow, particularly in relapsed or refractory disease. These patterns reflect the clinically aggressive behavior of MGZL compared with PMBL or CHL [13]. Reported epidemiologic differences across series largely reflect historical inclusion of extramediastinal gray zone lymphomas. When analysis is restricted to mediastinal cases, MGZL is characterized by younger age at diagnosis, predominantly early-stage disease, and a high frequency of bulky mediastinal involvement [4,13,14,15].

## 4. Pathology and Immunophenotype

Historically, GZL has been used to describe a heterogeneous group of lymphomas exhibiting varying degrees of morphologic and immunophenotypic discordance, positioned along the spectrum between classical CHL and PMBL [3]. In these cases, tumor cells are typically more numerous than in CHL and may resemble centroblastic or immunoblastic cells seen in PMBL; however, they are often larger, more pleomorphic, and display mixed cytologic features. Reed–Sternberg (RS)-like or lacunar cells may be present but are usually of intermediate size and show less prominent eosinophilic nucleoli compared with classical RS cells [4,8,16]. In contrast to CHL, the inflammatory background—particularly eosinophil- or plasma cell–rich infiltrates—is generally less conspicuous, while necrosis is uncommon or only mild when present [3,8,16]. Architectural patterns are frequently ill-defined, although nodular growth or coarse fibrosis may be observed in a subset of cases [4,16].

Importantly, many of these morphologic descriptions originate from historical GZL cohorts, which often included non-mediastinal and/or EBV-positive cases. Therefore, while these features illustrate the morphologic continuum between CHL and PMBL, they should be interpreted with caution when applied to strictly defined MGZL as currently recognized by WHO-HEMA5/ICC classifications.

### 4.1. B-Cell Markers and Transcription Factors

In MGZL, B-cell markers, including one or more of CD20, CD79a, and PAX5, are typically expressed at least focally, although their intensity and distribution may be variable [8]. Expression of B-cell-associated transcription factors such as BOB.1 and OCT-2 is likewise heterogeneous.

In cases with more centroblastic or PMBL-like morphology, CD20 expression may be weak or absent, whereas CD30 is frequently positive and CD15 may also be expressed [8,17]. Conversely, subsets of tumors containing abundant Reed–Sternberg-like cells may show strong expression of B-cell transcription factors (including PAX5, BOB.1/BCL6, and OCT-2), while lacking CD45 and often CD15 expression [4,8,16].

Importantly, these immunophenotypic patterns reflect the biological continuum between PMBL and CHL and should be interpreted in the context of strictly defined, EBV-negative mediastinal MGZL, as outlined in current WHO-HEMA5/ICC classifications.

### 4.2. Activation Markers and Additional Immunophenotypic Features

CD30 expression is observed in the majority of MGZL cases, although staining intensity is often weak or heterogeneous [8,16]. CD15 expression is variable, ranging from negative to moderate or strong positivity depending on morphologic features. Additional markers associated with mediastinal B-cell lymphomas, including CD23 and MAL, may be detected in subsets of cases [8,16].

Overexpression of PD-L1 and PD-L2 is common and has been linked to recurrent genetic alterations involving CIITA and CD274 (9p24.1), as demonstrated by fluorescence in situ hybridization in several studies [18]. These findings provide a biological rationale for immune checkpoint inhibition; however, their prevalence and predictive value in strictly defined MGZL are derived largely from small or retrospective cohorts.

EBER-positive cases have been infrequently reported in historical gray zone lymphoma series; however, under current WHO-HEMA5 and ICC criteria, EBV-positive lymphomas are explicitly excluded from the diagnosis of MGZL [19].

### 4.3. Proposed Morphophenotypic Subgroups

Sarkozy and colleagues proposed a classification of four morphophenotypic subgroups spanning a spectrum from CHL-like (groups 0–1) to PMBL-like (groups 2–3), based on architectural patterns, fibrosis, and immunophenotypic features [14,16]. While conceptually useful for illustrating the biologic continuum between CHL and PMBL, this subclassification is limited by its subjective criteria and the inclusion of non-mediastinal and EBV-positive cases, which are no longer encompassed within the contemporary WHO-HEMA5/ICC definition of MGZL [1,2]. Accordingly, this framework should be regarded as historically informative rather than diagnostically prescriptive, and its application to current MGZL practice requires caution.

In general, the immunophenotypic profile of MGZL lies intermediate between CHL and PMBL, with variable expression of B-cell markers, nearly universal expression of CD30, and variable CD15 expression. This immunophenotypic “gray zone” adds to the diagnostic challenge and supports MGZL as a distinct entity [18,20].

## 5. Molecular and Genetic Features

MGZL shares several molecular and genetic features with both PMBL and CHL, supporting its position as a biological intermediate between these entities. However, accumulating molecular data suggest that MGZL also harbors distinctive alterations, supporting its recognition as a separate entity within contemporary classification systems. Importantly, most available molecular studies are based on small or retrospective cohorts, and findings should therefore be interpreted with appropriate caution [21].

### 5.1. Molecular Features Overlapping with CHL and PMBL

Both PMBL and CHL frequently exhibit recurrent chromosomal gains involving the REL locus (2p16.1) and the JAK2/CD274/PDCD1LG2 region (9p24.1), resulting in overexpression of PD-L1 and PD-L2 and constitutive activation of the JAK/STAT signaling pathway [16,21]. Alterations affecting the CIITA locus (16p13.3) and dysregulation of antigen presentation pathways, including downregulation of MHC class II expression, are also commonly observed.

Similar genetic alterations have been reported in MGZL, supporting the concept of a molecular continuum across PMBL, MGZL, and CHL [16,21]. Nevertheless, these findings are largely derived from comparative analyses or mixed gray zone cohorts, and their specificity for strictly defined mediastinal, EBV-negative MGZL requires further validation.

### 5.2. Epigenetic and Gene Expression Profiles

Genome-wide methylation studies have demonstrated that MGZL exhibits an epigenetic profile intermediate between CHL and PMBL, while remaining distinct from DLBCL NOS [4,20]. Within this framework, hypomethylation of HOXA5 has been reported in MGZL and proposed as a potential distinguishing feature [4]. However, this observation originates from limited cohorts and should be regarded as hypothesis-generating, pending confirmation in larger, independently validated MGZL series.

Additional differentially expressed genes, including MMP9, EPHA7, and DAPK1, have been implicated in molecular clustering analyses aiming to discriminate MGZL from CHL and PMBL [21]. While these expression patterns provide insight into disease biology, their diagnostic or prognostic utility in routine clinical practice remains unproven.

### 5.3. Copy Number Alterations and Structural Variants

Copy number gains involving the REL locus (2p16.1) and MYC (8q24) have been reported in approximately one-third and one-quarter of MGZL cases, respectively [20]. These alterations may contribute to the relatively aggressive clinical behavior observed in some MGZL patients compared with PMBL or CHL.

In conjunction with 9p24.1 amplifications, these findings offer a potential biological rationale for sensitivity to immune checkpoint inhibition. However, direct associations between specific copy number alterations and clinical response in MGZL remain incompletely defined, given the limited size and heterogeneity of available cohorts [22,23].

### 5.4. Mutational Landscape

Next-generation sequencing studies have identified recurrent mutations in MGZL affecting key signaling pathways, including JAK/STAT, NF-κB, and genes involved in antigen presentation [2,24]. While these mutations further support the biological overlap between MGZL, PMBL, and CHL, their frequency, clonal architecture, and therapeutic relevance in strictly defined MGZL remain to be clarified. Future studies incorporating larger multicenter cohorts, uniform diagnostic criteria, and orthogonal validation methods will be required to determine the clinical significance of these alterations.

Collectively, these molecular alterations implicate the JAK/STAT, NF-κB, and antigen presentation pathways in the pathobiology of MGZL. Notably, cases displaying a gray zone lymphoma phenotype but not fulfilling the current diagnostic definition of mediastinal involvement (historically categorized as GZL) exhibit a distinct mutational landscape, with higher frequencies of TP53 (39%), BCL2 (28%), and BIRC6 (22%) mutations, as well as more frequent BCL2 and BCL6 rearrangements [9,25]. This molecular dissociation supports the current ICC and WHO-HEMA5 restriction of the MGZL category to EBV-negative lymphomas arising in the mediastinum [14].

### 5.5. Tumor Microenvironment and Epigenetic Plasticity

Gene expression profiling analyses further highlight the tumor microenvironment as an important component of MGZL biology. Comparative studies suggest that MGZL is characterized by enrichment of regulatory macrophage signatures and differential expression of immune checkpoint molecules, including PD-1, PD-L1, and LAG3, when compared with related mediastinal lymphomas [26]. These findings are consistent with an immune-rich microenvironment and provide a biological rationale for immune-targeted therapeutic approaches.

In addition, high-coverage sequencing has revealed a pronounced subclonal architecture in MGZL, with recurrent mutations affecting genes such as SOCS1, B2M, and TNFAIP3 arising in distinct subclones under therapeutic selection [2,13]. This clonal heterogeneity, together with epigenetic plasticity, may contribute to treatment resistance and disease relapse; however, the clinical implications of these observations remain to be fully elucidated.

## 6. Comparison of MGZL with PMBL and CHL

MGZL occupies a biological and clinical continuum between PMBL and CHL, particularly the nodular sclerosis subtype. While all three entities share origin from thymic B cells, they diverge in their morphology, immunophenotype, molecular characteristics, and clinical presentation [4,17,27,28]. These comparative features are summarized in Table 1.

## 7. Treatment Approaches

**Figure 1:**
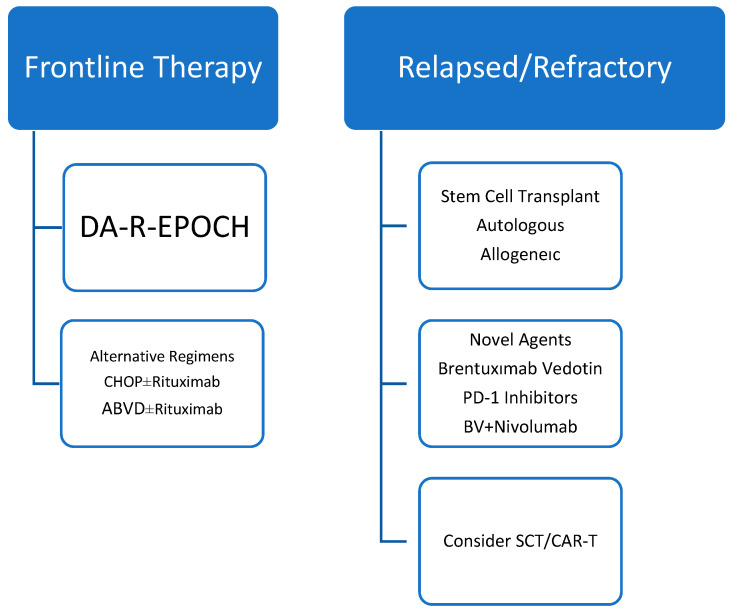
Evidence-graded treatment algorithm for MGZL. Treatment recommendations are primarily based on retrospective series and expert consensus. No randomized prospective trials have been conducted in strictly defined MGZL. Evidence levels and key caveats are indicated to guide clinical interpretation.

The management of MGZL remains challenging due to its rarity, biological heterogeneity, and the absence of standardized therapeutic guidelines. Owing to its clinicopathologic overlap with both PMBL and CHL, treatment strategies for MGZL have historically been extrapolated from these entities. However, available data suggest that outcomes in MGZL are heterogeneous and cannot be directly equated with those observed in PMBL or CHL [6]. An evidence-graded overview of current treatment approaches is summarized in Figure 1.

### 7.1. Frontline Therapy

Evidence guiding frontline therapy in MGZL is derived almost exclusively from retrospective series and population-based analyses, frequently including small patient numbers and, in older studies, heterogeneous gray zone cohorts. To date, no randomized prospective trials have been conducted specifically in strictly defined, EBV-negative mediastinal MGZL.

A population-based analysis from the National Cancer Database including 1047 patients diagnosed with gray zone lymphoma reported improved overall survival with combined-modality treatment compared with chemotherapy alone (hazard ratio 0.54, *p* = 0.005) [25]. However, the study lacked detailed information regarding salvage therapy, transplantation, and contemporary diagnostic criteria, limiting definitive conclusions regarding optimal treatment sequencing.

Dose-adjusted EPOCH-R (DA-EPOCH-R) has been among the most extensively studied regimens in MGZL. Early series from the National Institutes of Health reported a 5-year event-free survival (EFS) of 62% and overall survival (OS) of 74% in untreated MGZL patients [20]. While these outcomes appeared superior to those achieved with R-CHOP in historical comparisons, they remained inferior to results reported in PMBL treated with DA-EPOCH-R, underscoring the distinct biology of MGZL and the limitations of cross-entity comparisons [6,29].

Alternative regimens, including CHOP ± rituximab and ABVD ± rituximab, have been evaluated in small retrospective cohorts. In the series by Pilichowska et al., overall response rates were 65% for CHOP-based therapy and 60% for ABVD-based therapy, compared with 70% for DA-EPOCH-R [17]. Progression-free survival was inferior with ABVD-based approaches, suggesting limited disease control in this setting [29]. These findings highlight the trade-off between tolerability and efficacy in MGZL, though firm conclusions are constrained by limited sample sizes.

More intensive regimens such as escBEACOPP and ACBVP have been explored in selected European cohorts, demonstrating encouraging 3-year EFS (73–74%) and OS (86–94%) [14]. However, these outcomes likely reflect patient selection bias, with treatment generally restricted to younger, fit individuals able to tolerate substantial toxicity.

Outside the context of DA-EPOCH-R, novel dose-intensified strategies aiming to preserve efficacy while mitigating toxicity are emerging. Notably, R-COMP-DI, incorporating non-pegylated liposomal doxorubicin (Myocet™), was recently reported by Picardi et al. in patients with PMBL and MGZL [30]. In this retrospective series, six cycles of R-COMP-DI achieved end-of-treatment ^18^F-fluorodeoxyglucose positron emission tomography negativity in 93% of patients overall and in all MGZL cases. Importantly, treatment was associated with an acceptable safety profile, with no treatment-related hospitalizations and only limited grade ≥ 3 toxicities, primarily manageable hematologic and gastrointestinal events, despite dose-intensified anthracycline exposure.

Beyond its cytotoxic effects, liposomal doxorubicin may exert biologically relevant effects on the tumor microenvironment. Non-pegylated liposomal formulations are preferentially taken up by tumor-associated macrophages, a cell population enriched in both PMBL and MGZL and previously associated with adverse outcomes following conventional anthracycline-based therapy. Acting as a slow-release intratumoral reservoir, liposomal doxorubicin may enhance drug delivery while modulating macrophage-mediated immune suppression. In the study by Picardi et al., improved progression-free survival was observed in patients with higher intratumoral macrophage content treated with R-COMP-DI, suggesting a potential microenvironment-directed therapeutic benefit. Nevertheless, given the small sample size and retrospective design, these findings should be regarded as hypothesis-generating and warrant validation in prospective studies [30].

### 7.2. Safety and Tolerability of Frontline Immunochemotherapy

While intensive immunochemotherapy has improved disease control in MGZL, treatment-related toxicity remains a key determinant of regimen selection. Dose-dense and dose-adjusted regimens, particularly DA-EPOCH-R, are consistently associated with high rates of treatment-emergent adverse events. Grade ≥ 3 hematologic toxicities, including neutropenia, anemia, and thrombocytopenia, are frequently reported and often necessitate growth factor support, thereby increasing the risk of infectious complications [29]. Hospitalization for treatment-related toxicity is not uncommon, particularly during early cycles or at higher dose levels.

Less intensive regimens such as CHOP ± rituximab generally demonstrate a more favorable safety profile, with lower rates of severe myelotoxicity, but may provide inferior disease control compared with dose-intensive approaches [4]. Conversely, highly intensive regimens including escBEACOPP and ACBVP have achieved promising response rates at the expense of substantial myelotoxicity, infectious complications, and overall treatment burden, restricting their use to carefully selected, fit patients [29]. An evidence-graded treatment approach for mediastinal gray zone lymphoma is outlined in Table 2.

Overall, available safety data in MGZL are heterogeneous and derived from retrospective analyses, precluding definitive comparisons among regimens. A comparative overview of efficacy and toxicity profiles of frontline immunochemotherapy approaches in MGZL is provided in Table 3.

### 7.3. Role of Radiotherapy

The role of consolidative radiotherapy (RT) in MGZL has not been definitively established, largely owing to the rarity of the disease and the absence of prospective randomized studies. Nevertheless, RT has been employed in selected clinical scenarios, most commonly as consolidation following frontline immunochemotherapy in patients with bulky mediastinal disease at diagnosis or persistent residual mediastinal masses on post-treatment imaging [31].

Across published retrospective series, the indication for RT has typically been based on residual lymphadenopathy detected on post-therapy Computed Tomography (CT) or ^18^F-fluorodeoxyglucose positron emission tomography, most frequently located in the anterior mediastinum. When detailed, irradiated residual nodes were generally part of the initial bulky mediastinal mass, often measuring >7–10 cm at baseline, with variable degrees of size reduction following chemotherapy.

Radiation fields have predominantly consisted of involved-field radiotherapy (IFRT) in earlier reports and involved-site radiotherapy (ISRT) in more recent series, reflecting the transition toward modern radiation techniques aimed at minimizing exposure of adjacent critical organs, including the heart and lungs. Reported total radiation doses most commonly ranged between 30 and 36 Gy delivered in conventional fractions.

Radiotherapy was generally initiated 3–6 weeks after completion of immunochemotherapy, allowing for hematologic recovery. However, reporting of key parameters—including residual nodal size, radiation volume, and exact timing of RT initiation—has been inconsistent across studies, precluding firm conclusions regarding optimal RT indications or sequencing in MGZL [5,30,31,32].

Overall, while consolidative radiotherapy appears to be commonly used in patients with bulky or residual mediastinal disease, its impact on long-term outcomes remains uncertain, and current practice is guided primarily by extrapolation from PMBL and CHL rather than MGZL-specific evidence. Prospective studies are needed to better define the role of RT in this rare entity. The characteristics of consolidative radiotherapy reported in mediastinal gray zone lymphoma are summarized in Table 4.

### 7.4. Response Assesment

Response assessment in MGZL generally follows principles applied to other FDG-avid lymphomas but requires specific caution because residual fibrotic mediastinal masses are common after treatment. Accordingly, ^18^F-FDG PET/CT is the preferred modality for both interim and end-of-treatment response evaluation, as metabolic assessment more reliably distinguishes viable disease from post-therapy fibrosis than anatomic imaging alone [32]. Most centers use the Deauville five-point scale to interpret residual FDG uptake, with scores 1–3 generally considered negative and scores 4–5 positive [32,33]. In MGZL, persistent mediastinal masses with low or absent metabolic activity frequently represent inactive disease, and residual mass alone should not be interpreted as treatment failure. In equivocal cases—particularly when FDG uptake is borderline or discordant with clinical findings—histologic confirmation by biopsy should be considered before escalation of therapy. In the limited MGZL-specific literature, PET-guided strategies have informed subsequent management, including the selective use of involved-field radiotherapy in patients with localized PET-positive residual disease [6]. Early metabolic response assessed by interim PET has also been explored as a potential risk-adaptation tool; however, prospective data in strictly defined MGZL remain sparse, and PET findings should always be interpreted in conjunction with baseline disease characteristics and clinical context [34].

### 7.5. Relapsed/Refractory Disease and Stem Cell Transplantation

Relapsed or refractory (R/R) MGZL has traditionally been managed with salvage chemotherapy regimens such as ICE, ESHAP, or gemcitabine-based combinations, frequently followed by autologous stem cell transplantation (ASCT) in chemosensitive patients. In larger retrospective series, 2-year OS was approximately 88% in patients undergoing ASCT, compared with 67% in those who did not, underscoring the importance of transplantation in eligible patients [12,32,35,36]. Allogeneic stem cell transplantation has been employed in selected cases, although data remain limited.

### 7.6. Novel Agents and Biologic Therapies

The frequent expression of CD30 and recurrent 9p24.1 alterations leading to PD-1/PD-L1 upregulation provide a strong biological rationale for the use of antibody–drug conjugates and immune checkpoint inhibitors in MGZL [37].

Brentuximab vedotin (BV) has demonstrated activity as a single agent in case reports and small series and has also been used as maintenance therapy following ASCT [29,37]. Beyond its direct anti-CD30 cytotoxicity, BV may exert immunomodulatory effects by depleting regulatory T cells, potentially enhancing checkpoint blockade.

PD-1 inhibitors, including nivolumab and pembrolizumab, have shown clinically meaningful activity in R/R MGZL, with response patterns resembling those observed in CHL and PMBL [37]. The combination of BV plus nivolumab was evaluated in the CheckMate 436 trial, which reported an overall response rate of 70% and a complete remission rate of 50%, with durable responses (median progression-free survival ~22 months) and a favorable safety profile [38]. Importantly, this regimen enabled a proportion of patients to proceed to stem cell transplantation, supporting its role as an effective bridge-to-transplant strategy.

Given the high prevalence of PD-L1 expression in MGZL, more intensive immune-based approaches have also been explored. In a small prospective cohort, a dose-dense chemotherapy backbone combined with dual immune checkpoint inhibition (nivolumab plus ipilimumab) demonstrated promising activity in heavily pretreated, biologically aggressive MGZL. Early responses were frequent, with an estimated 18-month progression-free survival of 86%, although treatment-related toxicity—particularly profound neutropenia and renal adverse events—was substantial. These findings should be regarded as hypothesis-generating and highlight the potential of immune-modulating strategies in selected high-risk patients [39].

Overall, emerging data support a central role for immunotherapy-based approaches in R/R MGZL; however, prospective studies are required to define optimal combinations, sequencing, and integration with transplantation. An overview of novel agents in R/R MGZL is provided in Table 5.

### 7.7. Emerging Strategies

Although clinical experience remains limited, CAR T-cell therapies and bispecific antibodies are being actively explored in MGZL, largely based on its biological overlap with other aggressive B-cell lymphomas. Given the pronounced subclonal heterogeneity and epigenetic plasticity observed in MGZL, integration of molecularly guided therapies and immunotherapy-based approaches represents a promising direction for future management [39].

Novel CD20 × CD3 bispecific antibodies, including epcoritamab, glofitamab, and mosunetuzumab, have demonstrated substantial efficacy in relapsed or refractory aggressive B-cell lymphomas and may offer a chemotherapy-free therapeutic option for heavily pretreated MGZL patients. By redirecting cytotoxic T cells toward malignant B cells, these agents have the potential to overcome chemotherapy resistance while leveraging the immune-rich tumor microenvironment characteristic of MGZL. However, clinical data specifically evaluating bispecific antibodies in MGZL remain scarce, with current evidence largely derived from small retrospective series or extrapolated from studies in primary mediastinal large B-cell lymphoma and diffuse large B-cell lymphoma [12,35,36,37,38,39].

Similarly, cellular therapies, including CD19- or CD30-directed CAR T-cell therapy, represent a potential salvage strategy for patients with multiply relapsed disease. Although MGZL-specific outcome data are limited, extrapolation from PMBL and other aggressive B-cell lymphomas supports consideration of these approaches in highly selected cases [12,35,36,37,38,39].

Collectively, these emerging strategies underscore a shift toward biologically driven, immune-based treatment paradigms in MGZL, while emphasizing the need for dedicated prospective studies to define their optimal integration into clinical practice for this rare entity.

## 8. Prognosis and Outcomes

MGZL is associated with inferior outcomes compared with both PMBL and CHL, reflecting its aggressive clinical behavior and the absence of standardized treatment strategies. Although complete remissions can be achieved with intensive frontline chemoimmunotherapy, long-term disease control remains suboptimal [4,16,17].

Across published series, 5-year OS for MGZL ranges between 40–60%, substantially lower than outcomes reported for PMBL (>85–90%) and CHL (>80–85%) in the modern treatment era [14,20,23]. Similarly, EFS remains inferior, with 5-year EFS rates of approximately 60% following DA-EPOCH-R, compared with >90% reported in PMBL treated with the same regimen [6].

Several factors have been associated with adverse prognosis in MGZL, including advanced stage disease, bulky mediastinal masses, and extranodal involvement at diagnosis [15]. Treatment-related variables also appear important, as ABVD-based regimens are consistently associated with inferior outcomes compared with CHOP- or EPOCH-based approaches [6,17,30]. In addition, discordance between morphologic features and underlying molecular profiles has been linked to poorer treatment response in some series, though these observations remain derived from retrospective analyses [4].

Patients with relapsed or refractory MGZL experience particularly poor outcomes, with median progression-free survival often less than 12 months following salvage chemotherapy alone. In this setting, ASCT is associated with improved survival in eligible patients, with reported 2-year OS approaching 85–90%, compared with inferior outcomes in non-transplanted patients [12,33]. Emerging data suggest potential activity of brentuximab vedotin and immune checkpoint inhibitors, although evidence remains limited to small cohorts and early-phase studies [36].

Overall, the consistently inferior survival of MGZL compared with PMBL and CHL underscores its biological distinctiveness and unmet clinical need, emphasizing the necessity for optimized, MGZL-specific treatment strategies.

## 9. Conclusions

MGZL is a rare but clinically significant B-cell lymphoma situated at the biological and clinical interface between CHL and PMBL. Under current WHO-HEMA5 and ICC frameworks, MGZL is restricted to EBV-negative lymphomas arising in the mediastinum, reflecting improved understanding of its distinct biology.

Despite increasing molecular and immunologic insights, MGZL remains an under-studied entity, and most therapeutic recommendations continue to be extrapolated from PMBL or other aggressive B-cell lymphomas. Diagnostic heterogeneity, limited prospective data, and the absence of standardized treatment algorithms contribute to outcomes that remain inferior to those achieved in PMBL or CHL. Although intensive frontline regimens such as DA-EPOCH-R have improved initial disease control, relapse rates remain substantial and long-term survival unsatisfactory for a proportion of patients.

Advances in molecular profiling and immune-based therapies, including brentuximab vedotin and immune checkpoint inhibitors, have expanded treatment options, particularly in the relapsed or refractory setting. However, available evidence is largely derived from small retrospective series, underscoring the need for caution in interpretation. Future progress in MGZL will depend on international collaborative prospective studies, integration of molecular diagnostics into routine practice, and development of biologically driven, risk-adapted treatment strategies. Ultimately, combining conventional chemoimmunotherapy with targeted and immune-based approaches may offer the greatest potential to improve outcomes in this diagnostically and therapeutically challenging lymphoma.

## Figures and Tables

**Table 1:** Clinicopathologic comparison of MGZL, PMBL, and CHL.

Feature	PMBL	CHL (NS)	MGZL
Gender predilection	Female	Female	Male predominance
Age	30s	20–30s	30–40s
Morphology	Sheets of large cells, fibrosis	Lacunar RS cells, nodular sclerosis, inflammatory	Transitional, pleomorphic RS-like cells, less inflammation
B-cell markers	Strong (CD20, PAX5, OCT-2, BOB.1)	Weak/absent	Variable (≥1 marker usually)
CD30	Variable, weaker	Strong, diffuse	Usually positive
CD15	Negative	Positive (~75%)	Variable
CD45	Positive	Negative	Variable
Molecular profile	9p24.1 (JAK2/PD-L1/PD-L2), REL	9p24.1 (JAK2/PD-L1/PD-L2)	Intermediate + unique (HOXA5, MYC)
CD20	Strong	Negative/weak	Variable (often reduced)
PAX5	Strong	Weak	Variable (weak to moderate
CD79a/OCT-2/BOB.1	Strong Positive	Usually, absent Negative	Variable/heterogeneous Often variable; may be positive in RS-like forms
MAL	Positive	Negative	Often positive
PD-L1/PD-L2	Positive (9p24.1 amp)	Variable	Frequently positive
EBER	Rare	Rare	Occasionally reported (excludes MGZL under ICC/WHO-HEMA5

Abbreviations: CHL (NS subtype):classic Hodgkin lymphoma noduler sclerosis subtype, PMBL: Primary Mediastinal (Large) B-cell Lymphoma, MGZL: Mediastinal Gray Zone Lymphoma, PAX5: Paired box protein Pax-5, OCT-2: Octamer-binding Transcription Factor 2, BOB.1: B cell Oct binding protein 1, REL: v-rel avian reticuloendotheliosis viral oncogene homolog, HOXA5: Homeobox A5, RS-like: Reed–Sternberg like, MAL: Myelin and Lymphocyte Protein, EBER: Epstein–Barr virus-encoded small RNAs.

**Table 2:** Evidence-graded treatment approach in MGZL.

Clinical Setting	Recommended Approach	Level of Evidence	Key Caveats
Frontline therapy	DA-EPOCH-R	Retrospective cohorts	Small patient numbers; mixed historical GZL cohorts; outcomes not directly comparable to PMBL
	CHOP ± rituximab	Retrospective cohorts	Lower toxicity but potentially inferior disease control
	ABVD ± rituximab	Retrospective cohorts	Inferior PFS compared with CHOP-based regimens
Relapsed/refractory disease	Salvage chemotherapy → ASCT	Retrospective series	Limited MGZL-specific data; extrapolated from DLBCL/PMBL
	Allogeneic SCT	Case series	Selected patients only; higher toxicity
Novel agents	Brentuximab vedotin	Small series/case reports	CD30 expression variable; limited durability
	PD-1 inhibitors (±BV)	Small cohorts	Rationale based on 9p24.1 alterations; MGZL-specific data limited
Advanced salvage	CAR-T/SCT-based cellular therapy	Extrapolated evidence	Data derived from PMBL/DLBCL; MGZL-specific outcomes scarce

Abbreviations: DA-EPOCH-R: dose-adjusted etoposide, prednisone, vincristine (Oncovin), cyclophosphamide, doxorubicin plus rituximab, CHOP: cyclophosphamide, doxorubicin, vincristine (Oncovin), and prednisone, ABVD: doxorubicin (Adriamycin), bleomycin, vinblastine, and dacarbazine, PFS: progression-free survival, ASCT: autologous stem cell transplantation.

**Table 3:** Safety profile of frontline immunochemotherapy regimens in mediastinal gray zone lymphoma.

Regimen	Dose Strategy	Reported Grade ≥3 Toxicities	Other Safety Considerations
DA-EPOCH-R	Dose-adjusted, dose-dense	Neutropenia, anemia, thrombocytopenia, febrile neutropenia, infections	Frequent need for G-CSF; hospitalization not uncommon
CHOP ± Rituximab	Standard dose	Lower incidence of severe hematologic toxicity	Better tolerability but inferior disease control
escBEACOPP	Highly dose-intensive	Severe myelotoxicity, infections	High treatment-related morbidity; limited to fit patients
ACBVP	Dose-intensive	Grade ≥3 cytopenias, infections	Increased toxicity limits broad applicability
R-COMP-DI (liposomal doxorubicin)	Dose-intensified	Mainly hematologic; low incidence of severe non-hematologic toxicity	No reported hospitalization; favorable cardiac safety profile

Abbreviations: DA-EPOCH-R: dose-adjusted etoposide, prednisone, vincristine (Oncovin), cyclophosphamide, doxorubicin plus rituximab, CHOP: cyclophosphamide, doxorubicin, vincristine (Oncovin), and prednisone, escBEACOPP: escalated bleomycin, etoposide, doxorubicin (Adriamycin), cyclophosphamide, vincristine (Oncovin), procarbazine, and prednisone, ACBVP: doxorubicin (Adriamycin), cyclophosphamide, bleomycin, vincristine (Oncovin), and prednisone, R-COMP-DI: rituximab, cyclophosphamide, vincristine, and prednisone combined with dose-intensified non-pegylated liposomal doxorubicin.

**Table 4:** Characteristics of consolidative radiotherapy reported in mediastinal gray zone lymphoma.

Parameter	Commonly Reported Characteristics
Target site	Residual mediastinal lymph nodes (typically anterior mediastinum)
Residual nodal size	Often corresponding to initial bulky disease (>7–10 cm at diagnosis)
Imaging basis	Post-treatment CT or PET/CT
Radiotherapy volume	IFRT or ISRT
Total dose	30–36 Gy
Timing after chemotherapy	Usually 3–6 weeks after last cycle

Abbreviations: IFRT: involved-field radiotherapy, ISRT: involved-site radiotherapy, Gy: gray.

**Table 5:** Activity of novel agents in R/R MGZL.

Therapy	ORR	CR	Median PFS	Comments
Brentuximab Vedotın (BV)	Effective in case series	Variable (small numbers)	Not well defined	Also used as ASCT maintenance; immunomodulatory effect
PD-1 Inhibitors (Nivolumab, Pembrolizumab)	Clinical activiy reported in R/R MGZL	Variable (case-based)	Not well defined	Responses resemble CHL/PMBL
BV + Nivolumab	70%	50%	22 months	Durable responses, favorable safety; bridge to SCT

Abbreviations: ORR: Overall Response Rate, CR: Complete Response, PFS: Progression-Free Survival, ASCT: Autologous Stem Cell Transplantation, R/R: Relapsed/Refractory, SCT: Stem Cell Transplantation, CHL: Classical Hodgkin Lymphoma, PMBL: Primary Mediastinal (Large) B-cell Lymphoma.

## Data Availability

No new data were created or analyzed in this study. Data sharing is not applicable to this article.
